# Microrna-130a Downregulates HCV Replication through an atg5-Dependent Autophagy Pathway

**DOI:** 10.3390/cells8040338

**Published:** 2019-04-10

**Authors:** Xiaoqiong Duan, Xiao Liu, Wenting Li, Jacinta A. Holmes, Annie J. Kruger, Chunhui Yang, Yujia Li, Min Xu, Haiyan Ye, Shuang Li, Xinzhong Liao, Qiuju Sheng, Dong Chen, Tuo Shao, Zhimeng Cheng, Batul Kaj, Esperance A. Schaefer, Shilin Li, Limin Chen, Wenyu Lin, Raymond T. Chung

**Affiliations:** 1 Institute of Blood Transfusion, Chinese Academy of Medical Sciences and Peking Union Medical College, Chengdu 610052, China; xiaoqiongduan@yahoo.com (X.D.); yangchunhui@ibt.pumc.edu.cn (C.Y.); lily83630@163.com (Y.L.); xumin@ibt.pumc.edu.cn (M.X.); haiyan_ye0720@163.com (H.Y.); alyssa@bjmu.edu.cn (S.L.); liaoxzxy@163.com (X.L.); shilin-li@hotmail.com (S.L.); 2 Liver Center and Gastrointestinal Division, Department of Medicine, Massachusetts General Hospital, Harvard Medical School, Boston, MA 02114, USA; scnydxlx@126.com (X.L.); wtl9911002@163.com (W.L.); jacinta_holmes@hotmail.com (J.A.H.); annie.kruger@gunet.georgetown.edu (A.J.K.); qq_agnes@163.com (Q.S.); gzbobsums2004@126.com (D.C.); TSHAO@mgh.harvard.edu (T.S.); ZCHENG3@mgh.harvard.edu (Z.C.); BKAJ@mgh.harvard.edu (B.K.); eschaefer@mgh.harvard.edu (E.A.S.); Chung.Raymond@mgh.harvard.edu (R.T.C.); 3 College of Animal Science and Technology, Southwest University, Chongqing 400715, China; 4 Department of Infectious Disease, Anhui Provincial Hospital, Anhui Medical University, Hefei 230000, China; 5 Department of Gastroenterology, St Vincent’s Hospital, Fitzroy, VIC 3065, Australia

**Keywords:** miR-130a, autophagy, autophagy-related genes 5 (ATG5), ATG12, hepatitis C virus (HCV), interferon stimulated gene (ISG)

## Abstract

We previously identified that miR-130a downregulates HCV replication through two independent pathways: restoration of host immune responses and regulation of pyruvate metabolism. In this study, we further sought to explore host antiviral target genes regulated by miR-130a. We performed a RT² Profiler™ PCR array to identify the host antiviral genes regulated by miR-130a. The putative binding sites between miR-130a and its downregulated genes were predicted by miRanda. miR-130a and predicted target genes were over-expressed or knocked down by siRNA or CRISPR/Cas9 gRNA. Selected gene mRNAs and their proteins, together with HCV replication in JFH1 HCV-infected Huh7.5.1 cells were monitored by qRT-PCR and Western blot. We identified 32 genes that were significantly differentially expressed more than 1.5-fold following miR-130a overexpression, 28 of which were upregulated and 4 downregulated. We found that ATG5, a target gene for miR-130a, significantly upregulated HCV replication and downregulated interferon stimulated gene expression. miR-130a downregulated ATG5 expression and its conjugation complex with ATG12. ATG5 and ATG5-ATG12 complex affected interferon stimulated gene (ISG) such as MX1 and OAS3 expression and subsequently HCV replication. We concluded that miR-130a regulates host antiviral response and HCV replication through targeting ATG5 via the ATG5-dependent autophagy pathway.

## 1. Introduction

Noncoding RNA plays an important role in viral infection and host immune responses [1]. MicroRNA (miRNA) is a class of small non-coding RNA that negatively regulates gene expression at the transcriptional or post-transcriptional level. We previously identified that miR-130a downregulates HCV replication through the expression of several host innate immunity related genes including type I IFN (IFNα/IFN β), ISG15, USP18 and MxA; and via the cellular pyruvate metabolic pathway [2,3]. Each miRNA can target multiple genes, and a single gene may be targeted by several miRNAs [4,5]. In this study, we sought to further explore other possible genes targeted by miR-130a. We performed a RT² Profiler™ PCR array to investigate the effect of miR-130a overexpression on 84 genes involved in the antiviral response pathway. We found that autophagy 5 (ATG5) expression was one of the most significantly downregulated genes in the context of miR-130a overexpression. Autophagy is an essential process that degrades proteins, damaged organelles and intracellular bacteria to maintain cell homeostasis [6,7]. The regulation of the autophagy process consists of more than 30 molecules, genes and proteins [8]. Several human viruses including Epstein-Barr virus (EBV), hepatitis B virus (HBV), hepatitis C virus, Sendai virus, and Dengue virus can induce autophagy to promote virus infection and replication [9,10,11]. HCV infects approximately 71 million people worldwide, and is a leading cause of liver cirrhosis and hepatocellular carcinoma (HCC) [12]. HCV infection activates the JAK-STAT pathway leading to the production of several hundred interferon stimulated genes (ISGs) that produce an antiviral state to restrict HCV spread within liver [1,3,13,14,15,16,17]. However, the biologic regulatory mechanisms by which miRNA regulate HCV infection through ISGs are not been well characterized. HCV infection also induces autophagy in vitro, as well as in liver biopsies from patients with chronic HCV [9,18]. The mechanisms by which autophagy promotes HCV replication are not well explored. Autophagy has been shown to be required for hepatitis C virus replication initiation, the downregulation of autophagy molecules is associated with reduced HCV replication [19]. Several studies have demonstrated that autophagy enhances HCV RNA replication by providing membrane for the HCV replication complex assembly [9,20,21]. Autophagy has also been found to affect HCV viral release [22,23]. Furthermore, autophagy also regulates HCV infection through disruption of the host antiviral response. Autophagy-related 5 (ATG5) is a key molecule in initiating autophagy through complexing with ATG12. The ATG5-ATG12 complex negatively regulates type I interferon (IFN) production in mouse embryonic fibroblasts (MEFs) to facilitate vesicular stomatitis virus replication [24]. ATG5 has also been shown to be essential for type I IFN production in plasmacytoid dendritic cells infected with vesicular stomatitis virus [25]. Knockdown of BCN1 or ATG7 increases IFN-α/β and the interferon-stimulated gene OAS1 expression in HCV-infected immortalized human hepatocytes [26]. Several miRNAs have been demonstrated to regulate autophagy, including miR-130a, which has been shown to regulate autophagy in endothelial progenitor cells and chronic lymphocytic leukemia cells [27,28]. However, the crosstalk between miR-130a, HCV infection and autophagy are still not well characterized. We therefore investigated the role of miR-130a downregulation on HCV replication through the ATG5-dependent autophagy pathway and on ISGs. 

## 2. Materials and Methods

### 2.1. Cells and Virus

Huh7.5.1 cells were grown in DMEM (Logan, UT, USA) supplemented with 10% fetal bovine serum (FBS) (GIBCO, Waltham, MA, USA) and 1% penicillin/streptomycin (Logan, UT, USA) at 37 °C in a 5% CO2 incubator. Huh7.5.1 cells were infected with genotype 2a JFH1 HCV at a multiplicity of infection (MOI) of 0.2 (JFH1 cells), as previously described [1,3]

### 2.2. MicroRNA Mimics, siRNAs, CRISPR/cas9 gRNAs, Plasmids and Transfection

miR-130a mimic, ATG5 siRNAs and their corresponding negative controls were purchased from GE Dharmacon (Lafayette, CO, USA) and transfected into cells using Lipofectamine® RNAiMAX reagent (Thermo Fisher Scientific, Waltham, MA USA). GenCRISPR gRNAs for miR-130a and ATG5 were purchased from GenScript USA Inc (Piscataway, NJ, USA). Protospacer sequences of CRISPR/cas9 against miR-130a or ATG5 were designed by CRISPR DESIGN (http://crispr.mit.edu/). The generated gRNA sequence was cloned in the pGS-gRNA-Neo vector. CRISPR/cas9 gRNAs were transfected into cells using Lipofectamine LTX with Plus^TM^ reagent (Thermo Fisher Scientific). G418 (Thermo Fisher Scientific) was added for selection of miR-130a or ATG5 knock down cells. The ATG5 plasmid and the corresponding empty vector were transfected into cells using Lipofectamine LTX reagent, as previously described [1,3].

### 2.3. RNA Isolation, Rt² Profiler™ Pcr Array and Gene Quantification

The RT² Profiler™ PCR array (PAHS-122ZA) for human antiviral responses was purchased from Qiagen (Gaithersburg, MD, USA). Total RNA was extracted using TRIzol reagent (Invitrogen, Carlsbad, CA, USA). The first strand cDNA was synthesized using ReverTra Ace® qPCR RT Master Mix with gDNA remover (Toyobo, Osaka, Japan). The cDNA was diluted with nuclease-free water, mixed with Power Up SYBR Green Master Mix (Thermo Fisher Scientific) and loaded into the 96-well RT² Profiler™ PCR Array plate. The confirmatory quantitative real time PCR (qRT-PCR) of the array hits was performed using the Bio-Rad CFX-96. The mRNA level of each gene was calculated using ∆∆Ct method, normalized to GAPDH. Primers for qRT-PCR are listed in Table 1.

### 2.4. Western Blotting and Antibodies

Protein samples were prepared using RIPA buffer containing a protease inhibitor cocktail (Sigma Life Science and Biochemicals, St. Louis, MO, USA). Proteins were separated by SDS-PAGE and transferred to a PVDF membrane as previously described [3]. Primary antibodies included Rabbit anti-human ATG5 (Cell Signaling technology, Beverly, MA, USA), mouse anti-HCV core (Fisher Scientific, Pittsburgh, PA, USA), and mouse anti-β-actin (Sigma, St. Louis, MO, USA). The secondary antibodies were horseradish peroxidase (HRP)-conjugated enhanced chemiluminescence (ECL) donkey anti-rabbit IgG and HRP-conjugated ECL sheep anti-mouse IgG (GE Healthcare Biosciences, Pittsburgh, PA, USA). The blots were subjected to chemiluminescence assay using the Amersham ECL Western blotting detection kit (GE Healthcare Biosciences, Pittsburgh, PA, USA).

### 2.5. Luciferase Reporter Assay

The putative binding sites between the ATG5 3’UTR and miR-130a seed sequence was predicted by MiRanda. Wide-type and mutated ATG5 3’UTR harboring miR-130a binding sites was constructed into the 3’UTR of the *Renilla* luciferase gene in the dual-luciferase reporter vector pmiR-RB-Report™ (Ribobio, Guangzhou, China). The vector has a firefly luciferase gene (luc) as an internal control. The plasmids were co-transfected with miR-130a mimic or the negative control into 293T cells. Firefly and Renilla luciferase activities were measured by use of a Promega dual-luciferase reporter assay at 48 h post-transfection. The Renilla luciferase/firefly luciferase activity ratio was calculated to determine the binding between the cloned 3’UTR and miR-130a. 

### 2.6. Statistical Analysis

Data analyses were performed using a 2-tailed Student’s t-test. Data are expressed as mean ± standard deviation of at least three biologic replicates, unless stated otherwise. In all analyses, * represents *p* < 0.05, ** represents *p* < 0.01 and *** represents *p* < 0.001 for indicated comparisons.

## 3. Results

### 3.1. miR-130a Regulates Host Antiviral Response Genes

**Figure 1:**
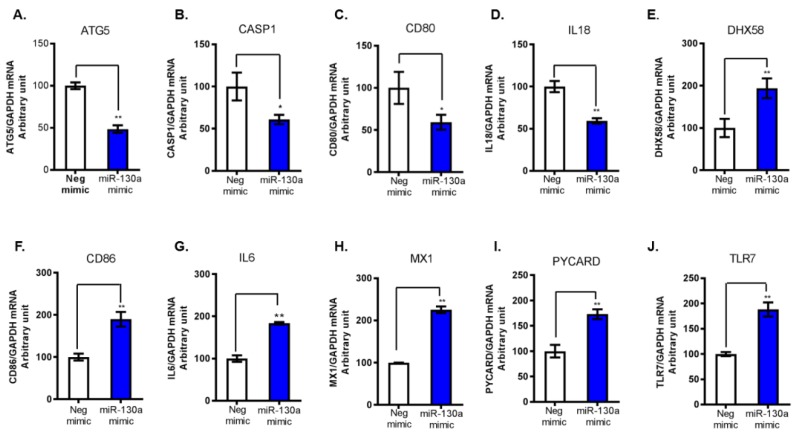
Top 10 dysregulated genes by miR-130a overexpression. We selected 10 PCR array identified genes for further validation by qPCR, the four downregulated genes (**A**–**D**) and the six-innate immunity related upregulated genes (**E**–**J**). mRNA levels of each gene were normalized to GAPDH mRNA yielding arbitrary units (fold change). Data are expressed as mean ± SD of three sample replicates. * *p* < 0.05, ** *p* < 0.01. (**A**–**D**) The four downregulated genes following miR-130a overexpression were confirmed by qPCR: (**A**) ATG5; (**B**) CASP1; (**C**) CD80; and (**D**) IL18. (**E**–**J**) The selected six upregulated genes following miR-130a overexpression: (**E**) DHX58; (**F**) CD86; (**G**) IL6; (**H**) MX1; (**I**) PYCARD; and (**J**) TLR7.

In our previous study, we found that miR-130a inhibits HCV replication by regulation of the expression of several host innate immunity related genes including type I IFN (IFNα/IFNβ), ISG15, USP18 and MxA [2]. To investigate the mechanism by which miR-130a regulates the innate immune response, we transfected miR-130a mimic or negative control to Huh7.5.1 cells. We performed a human antiviral response RT² Profiler™ PCR array (PAHS-122ZA). Of the 84 genes involved in human innate immune responses, 74 genes were detected, and 10 genes were undetectable in both control and miR-130a overexpressed cells. We identified 32 genes that were significantly differentially expressed more than 1.5-fold following miR-130a overexpression: 28 genes were upregulated and 4 genes were downregulated (Table 2). Pathway analyses of the upregulated genes revealed that virus recognition (DHX58, DDX58 TLR7 and TLR9), interferon production (IRAK1, MyD88, TBK1 and IRF5), and interferon-stimulated genes (MX1) were represented. The four downregulated genes were autophagy related 5 (ATG5), Caspase 1 (CASP1), Interleukin-18 (IL18) and CD80. We selected the top 6 upregulated genes and all four downregulated genes for further validation by qRT-PCR and confirmed the PCR array data (Figure 1).

### 3.2. ATG5 Is a Target Gene for miR-130a

**Figure 2:**
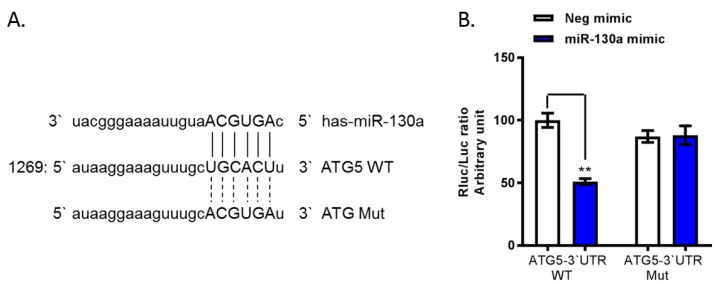
miR-130a target binding site on the 3’UTR of ATG5. (**A**) Putative binding site of the miR-130a seed sequence on the 3’UTR of ATG5. ATG5 with a mutation in the 3’UTR (ATG5 3’UTR-Mut) was constructed by compensatory mutagenesis. (**B**) The *Rluc/luc* ratio was significantly reduced following co-transfection of miR-130a mimic and the wild type ATG5 3’UTR, but not in the ATG4 3’UTR-Mut construct. Data are expressed as mean ± SD of three sample replicates. ** *p* < 0.01.

To determine whether the four downregulated genes are target genes for miR-130a, we used miRanda to predict the possible binding sites of the 3’UTR of these genes (ATG5, CASP1, IL18 and CD80) with the miR-130a seed sequence (AGUGCA). One possible binding site for the miR-130a seed sequence was observed in the 3’UTR of ATG5 (Figure 2A). Therefore, we cloned the 3’UTR of ATG5 downstream of the *Renilla* luciferase reporter gene in a pmiR-RB-Report^TM^ vector and co-transfected miR-130a mimic or the negative mimic control. We found the ratio of Rluc/luc was significantly decreased by 49% in miR-130a overexpression (mimic) cells compared to control cells (Figure 2B). In contrast, the mutated ATG5 3’UTR of the binding sites for miR-130a did not exert this inhibition (Figure 2B). These findings indicate that ATG5 is a target gene for miR-130a.

### 3.3. miR-130a Downregulates ATG5 Expression and HCV Replication

**Figure 3:**
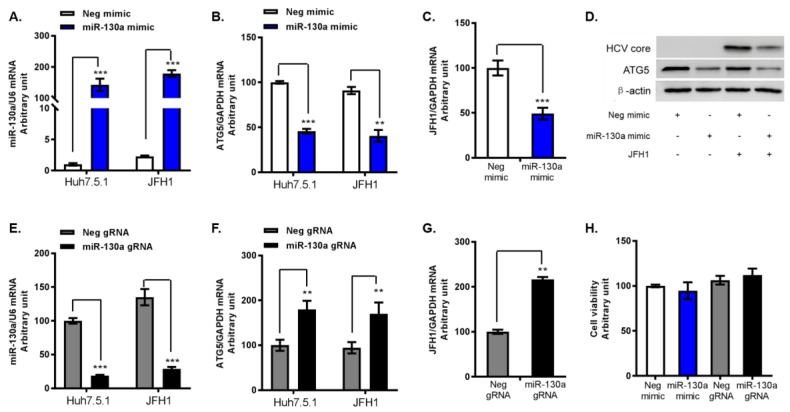
miR-130a regulates ATG5 expression and HCV replication. miR-130a mimic or miR-130a gRNA and their corresponding negative control were transfected to Huh7.5.1 cells and Huh7.5.1 cells infected with JFH1 HCV infection at a multiplicity of infection (MOI) of 0.2 (JFH1 cells). Total RNA or protein of the cells was harvested at 72 h post vector transfection and 48 h post JFH1 HCV infection. (**A**) Level of miR-130a overexpression; (**B**) miR-130a overexpression reduces ATG5 mRNA levels in Huh7.5.1 and JFH1 cells; (**C**) miR-130a overexpression inhibits HCV RNA expression in JFH1 cells; (**D**) miR-130a mimic reduces ATG5 and HCV core protein levels in Huh7.5.1 and JFH1 cells; (**E**) miR-130a gRNA knocks down miR-130a expression in Huh7.5.1 and JFH1 cells; (**F**) miR-130a gRNA increases ATG5 mRNA levels; (**G**) miR-130a gRNA promotes HCV RNA expression; and (**H**) miR-130a mimic and miR-130a gRNA transfection does not affect cell viability. Data are expressed as mean ± SD of three sample replicates. ** *p* < 0.01, and *** *p* < 0.001.

To further determine the regulatory effects of miR-130a on HCV replication and ATG5, we transfected miR-130a mimic and miR-130a gRNA into Huh7.5.1 cells with or without infected with JFH1. We again confirmed that miR-130a overexpression significantly reduced ATG5 mRNA levels by 55% and 42% in Huh7.5.1 and JFH1 infected Huh7.5.1 cells, respectively (Figure 3A,B). In addition, miR-130a overexpression significantly inhibited HCV RNA replication and HCV core expression (Figure 3C,D). Conversely, miR-130a knock down by miR-130a gRNA significantly increased ATG5 mRNA levels and promoted HCV RNA replication (Figure 3E–G). miR-130a overexpression and knock down did not affect cell viability (Figure 3H). These findings indicate that miR-130a regulates both ATG5 expression and HCV replication.

### 3.4. ATG5 Upregulates HCV Replication through Downregulation of ISG Expression

**Figure 4:**
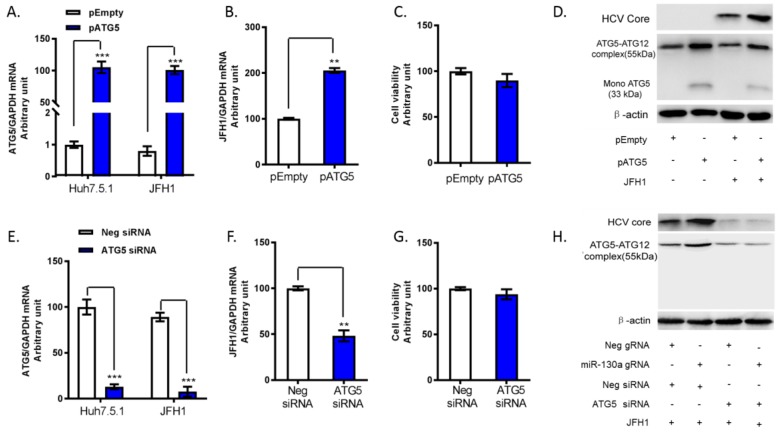
ATG5 upregulates HCV replication through conjugation complexing with ATG12. pATG5, pEmpty, Neg siRNA and ATG5 siRNA were transfected into Huh7.5.1 cells or miR-130a knock down cells. Total RNA or protein of the cells was harvested at 48 h post JFH1 HCV (JFH1 at 0.2 MOI) infection. The selected gene mRNAs were assessed by qPCR. The protein levels were monitored by Western blot. (**A**) Overexpression of pATG5 increases ATG5 mRNA levels compared to pEmpty; (**B**) overexpression of pATG5 promotes HCV RNA expression in JFH1 infected Huh7.5.1 cells compared to pEmpty; (**C**) overexpression of pATG5 does not affect cell viability; (**D**) overexpression of pATG5 increases both monomeric ATG5 and the ATG5-ATG12 complex protein levels and promotes HCV core levels compared to pEmpty; (**E**) ATG5 siRNA decreases ATG5 levels in Huh7.5.1 and JFH1 cells compared to Neg siRNA; (**F**) ATG5 siRNA inhibits HCV RNA levels compared to Neg siRNA; (**G**) ATG5 siRNA does not affect cell viability; and (**H**) ATG5 siRNA decreases ATG5 and HCV core protein levels. ATG5 siRNA abrogates the increase of ATG5 and HCV core in miR-130a gRNA knock down cells. Data are expressed as mean ± SD of at least three sample replicates. ** *p* < 0.01, and *** *p* < 0.001.

**Figure 5:**
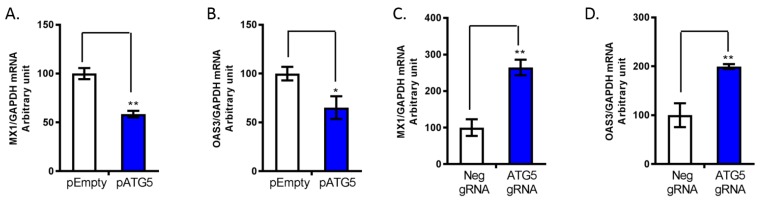
ATG5 downregulates classical interferon-stimulated gene expression. pATG5, pEmpty, Neg gRNA and ATG5 gRNA were transfected into Huh7.5.1 cells. The selected gene mRNAs were assessed by qPCR. (**A**) Overexpression of pATG5 decreases MX1 mRNA levels; (**B**) overexpression of pATG5 decreases OAS3 mRNA levels; (**C**) ATG5 gRNA increases MX1 mRNA levels; and (**D**) ATG5 gRNA increases OAS3 mRNA levels. Data are expressed as mean ± SD of at least three sample replicates. * *p* < 0.05, ** *p* < 0.01.

Next, we investigated the regulatory effect of ATG5 on HCV replication. We transfected ATG5 siRNA or ATG5 plasmid to knock down and overexpression of ATG5 in JFH1-infected Huh7.5.1 cells. As previously reported [24,29], ATG5 is covalently modified with a ubiquitin-like modifier to form ATG5-ATG12 complex. We confirmed that ATG5-ATG12 migrate as a complex in an SDS-gel. We found primary form of the ATG5 protein was the ATG5-ATG12 complex, which represents normal ATG5 protein function (Figure 4D). Overexpression of ATG5 increased HCV RNA and HCV core protein levels (Figure 4B,D). In contrast, knock down of ATG5 significantly inhibited HCV RNA and HCV core levels (Figure 4E–H). Furthermore, we confirmed that miR-130a knock down significantly increased ATG5 protein levels and HCV core expression. Silencing of ATG5 in miR-130a knock down cells abrogated the increase of HCV core protein (Figure 4H). We further assessed the expression of classical ISGs. We found that ATG5 overexpression reduced MX1 and OAS3 expression, while ATG5 knock down increased MX1 and OAS3 mRNA expression (Figure 5A–D). These findings indicate that ATG5 regulates HCV replication through classical ISGs such as MX1 and OAS3.

## 4. Discussion 

MicroRNAs play an important role in the regulation of virus infection and host immunity [30]. In our previous study, we found that miR-130a downregulates HCV replication through restoration of host immunity and through targeting PKLR [2,3]. In the present study, we further identified that miR-130a downregulates HCV infection via the ATG5 autophagy pathway and through regulation of antiviral response genes. Consistent with previous observations [2,3,30], several innate immunity antiviral genes including DHX58, TLR7 and MX1 were significantly increased after miR-130a overexpression, highlighting the critical role of miR-130a in host antiviral responses. Of the 84 differentially expressed genes involved in human innate immune responses identified on our PCR array, only four genes were downregulated by miR-130a overexpression. As miRNAs function through suppression of their target genes [31,32], we next investigated these four down regulated genes for potential binding sites for the miR-130a seed sequence. We subsequently identified that ATG5, an autophagy gene, is another target gene for miR-130a. 

Autophagy is a conserved catabolic process to maintain cellular hemostasis. Emerging evidence supports that miRNAs play a critical role in regulating autophagy [11,19,33]. miR-130a has been reported to regulate autophagy through targeting runt-related transcription factor 3 (Runx3) in endothelial progenitor cells [27]. miR-130a has also been found to inhibit autophagy by regulating ATG2B in lymphocytic leukemia cells [28]. Consistent with these reports, we demonstrated that miR-130a overexpression downregulates ATG5 and subsequently the ATG5-ATG12 protein conjugation complex. Furthermore, several studies have shown that autophagy positively regulates the HCV life cycle [21,22,23,34]. Knock down of ATG7 or Beclin1 inhibits the release of infectious HCV particles into the extracellular medium [22,23]. ATG5 has been reported to interact with HCV NS5B protein and has been utilized as a proviral factor during HCV infection [34]. Autophagosomes are double-membraned vesicles, the formation of which is controlled by the ATG5-ATG12 and LC3 complexes. The ATG5-12/16L1 complex was found to be required for HCV replication [21]. In our study, we found that miR-130a downregulates the ATG5-ATG12 protein conjugation complex. Overexpression of miR-130a reduced the ATG5-ATG12 protein complex, while knockdown of miR-130a increased the protein levels of the ATG5-ATG12 protein. Overexpression of ATG5 increased the protein level of the ATG5-ATG12 complex, as well as HCV replication. These findings further confirm that miR-130a downregulates HCV replication through the regulation of the ATG5-dependent autophagy pathway. In addition, to affect virus directly, autophagy has also been found to regulate viral replication through crosstalk with host immunity. For example, IFN-induced TDRD7 (tudor domain containing 7) inhibits paramyxovirus replication by inhibiting autophagy, and influenza A virus induced IFN-β expression is increased in ATG5 deficient cells [35]. In addition, ATG5 deficient mouse embryonic fibroblasts (MEFs) are resistant to vesicular stomatitis virus replication owing to hyperproduction of type I IFNs [24]. The ATG5–ATG12 protein conjugate complex negatively regulates the type I IFN pathway by direct association with the retinoic acid-inducible gene I (RIG-I) and IFN-β promoter stimulator 1 (IPS-1) through the caspase recruitment domains (CARDs) [24]. In our study, we observed the significant upregulation of several classic ISGs, including MX1 and OAS3, in miR-130a overexpression or ATG5 knock down cells, and reduction in MX1 and OAS3 expression with ATG5 overexpression. These findings indicate that miR-130a regulates HCV replication through the ATG5-dependent autophagy and subsequently on ISG expression.

## 5. Conclusions

**Figure 6:**
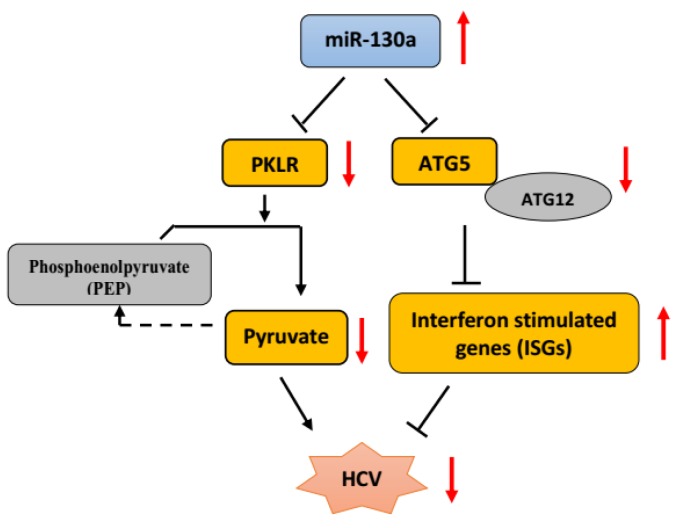
Proposed model for miR-130a downregulation of HCV infection. miR-130a targets two genes, PKLR and ATG5, and subsequently two independent pathways to downregulate HCV replication. One is through targeting PKLR to regulate the pyruvate metabolic pathway, and the other is through targeting ATG5 to regulate autophagy to regulate host antiviral interferon-stimulated genes (ISGs) (red arrows indicate up- or downregulation).

We conclude that miR-130a plays an important role in regulating HCV replication through targeting autophagy pathway genes, in addition to its previously identified role in regulating metabolism via PKLR. We found that miR-130a overexpression downregulated ATG5 expression and ATG5-ATG12 conjugation, which negatively regulated the interferon-stimulated gene expression. We confirmed that miR-130a inhibits HCV replication by restoring the innate immune response. We speculate that miR-130a regulates ATG5 and subsequently LC3 or Beclin-1 autophagy pathway. Further characterization of miR-130a regulates HCV replication through ATG5 and LC3 or Beclin-1 is warranted. We therefore propose a novel pathway by which miR-130a affects HCV infection through targeting of two independent genes, PKLR and ATG5. miR-130a targets PKLR to mediate pyruvate metabolism and ATG5 to regulate host antiviral response genes (Figure 6). Our data provide further understanding of the complex interplay between microRNA, autophagy, host antiviral response genes and HCV replication. These findings have important implications for the design and development of novel antiviral therapeutic applications for HCV infection, particularly in the small proportion of patients that may not respond to direct-acting antiviral therapy, as well as other, related IFN-sensitive viral infections. 

## Figures and Tables

**Table 1:** List of primer sequences used for qPCR validation.

Gene Name	Forward Primer	Reverse Primer
ATG5	TGTGCTTCGAGATGTGTGGTT	ACCAACGTCAAATAGCTGACTC
CASP1	CAGCCCTGGTGTGGTGTG	AAAATCCTTCTCTATGTGGGCTTTC
CD80	CACCTGGCTGAAGTGAC	GTCAGGCAGCATATCAC
IL18	CTTCCAGATCGCTTCCTCTC	TCAAATAGAGGCCGATTTCC
DHX58	GGGCCTCCAAACTCGATGG	TTCTGGGGTGACATGATGCAC
MX1	GTTTCCGAAGTGGACATCGCA	GAAGGGCAACTCCTGACAGT
OAS3	GAAGGAGTTCGTAGAGAAGGCG	CCCTTGACAGTTTTCAGCACC
PYCARD	TGGTCAGCTTCTACCTGGAG	CAGCCACTCAACGTTTGTGA
CD86	GGGCCGCACAAGTTTTGA	GCCCTTGTCCTTGATCTGAA
IL6	GACAACTTTGGCATTGTGG	ATGCAGGGATGATGTTCTG
TLR7	TCCTTGGGGCTAGATGGTTTC	TCCACGATCACATGGTTCTTTG
JFH1	TCTGCGGAACCGGTGAGTA	TCAGGCAGTACCACAAGGC
GAPDH	ACCTTCCCCATGGTGTCTGA	GCTCCTCCTGTTCGACAGTCA

**Table 2:** List of differentially expressed antiviral genes identified by PCR array.

Gene Name	Relative Expression *	Gene Name	Relative Expression
DHX58	4.27	MYD88	1.65
PYCARD	3.44	ISG15	1.63
TLR7	2.99	RELA	1.63
MX1	2.67	DDX58	1.62
CD86	2.38	TRADD	1.61
IL6	2.3	MAPK3	1.6
PYDC1	1.95	CASP10	1.58
MAPK14	1.95	CTSS	1.58
MAP3K1	1.95	MAPK1	1.56
IRAK1	1.95	TBK1	1.55
SPP1	1.9	IRF5	1.54
FADD	1.85	TLR9	1.53
JUN	1.83	IL18	0.54
NFKBIA	1.77	ATG5	0.55
CXCL11	1.69	CD80	0.65
IKBKB	1.66	CASP1	0.67

* Relative expression represents fold change of the tested gene normalized to GAPDH following miR-130a overexpression.
